# μ-Opioid Receptor Stimulation in the Nucleus Accumbens Increases Vocal–Social Interactions in Flocking European Starlings, *Sturnus Vulgaris*

**DOI:** 10.1523/ENEURO.0219-21.2021

**Published:** 2021-09-24

**Authors:** Alyse N. Maksimoski, Brandon J. Polzin, Sharon A. Stevenson, Changjiu Zhao, Lauren V. Riters

**Affiliations:** Department of Integrative Biology, University of Wisconsin-Madison, Madison, Wisconsin 53706

**Keywords:** flocking, mesolimbic reward pathway, nucleus accumbens, opioids, social behavior, songbirds

## Abstract

Social connections in gregarious species are vital for safety and survival. For these reasons, many bird species form large flocks outside the breeding season. It has been proposed that such large social groups may be maintained via reward induced by positive interactions with conspecifics and via the reduction of a negative affective state caused by social separation. Moreover, within a flock optimal social spacing between conspecifics is important, indicating that individuals may optimize spacing to be close but not too close to conspecifics. The μ-opioid receptors (MORs) in the nucleus accumbens (NAc) are well known for their role in both reward and the reduction of negative affective states, suggesting that MOR stimulation in NAc may play a critical role in flock cohesion. To begin to test this hypothesis, social and nonsocial behaviors were examined in male and female European starlings (*Sturnus vulgaris*) in nonbreeding flocks after intra-NAc infusion of saline and three doses of the selective MOR agonist d-Ala^2^-*N*-Me-Phe^4^-Glycol^5^-enkephalin (DAMGO). DAMGO in NAc dose-dependently increased singing behavior and facilitated social approaches while at the same time promoting displacements potentially used to maintain social spacing. These findings support the hypothesis that MORs in NAc promote social interactions important for group cohesion in nonsexual contexts and suggest the possibility that MORs in the NAc play a role in optimizing the pull of joining a flock with the push of potential agonistic encounters.

## Visual Abstract

**Figure:**
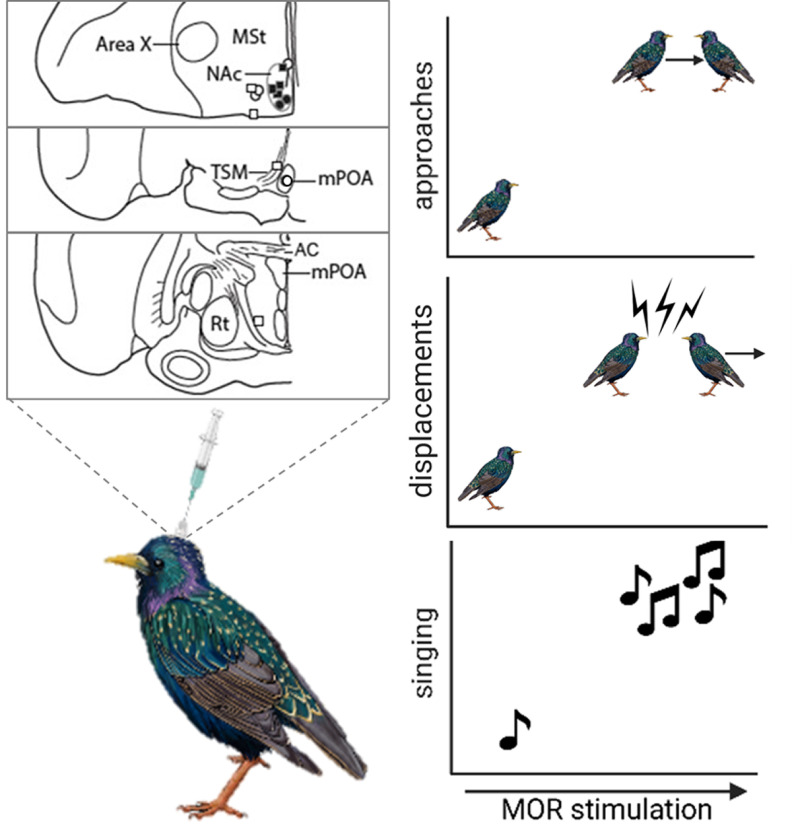

## Significance Statement

Social interactions with conspecifics are vital for safety and survival. Group cohesion in gregarious species may be maintained by social reward and reduced negative affect, yet underlying mechanisms have not been well studied. The μ-opioid receptors (MORs) in the nucleus accumbens (NAc) are strongly implicated in reward. Here, we demonstrate in flocks of European starlings that MOR stimulation in the NAc increases affiliative singing and approach behaviors as well as displacements. These results demonstrate a role for MORs in the NAc in both affiliative behaviors and behaviors used to maintain social spacing. Findings suggest that opioids in the NAc may play a role in optimizing the pull of joining a flock with the push of potential agonistic encounters.

## Introduction

In many species, social connections outside a breeding context are vital for both safety and survival. For instance, schooling allows teleost fish to avoid predators (Magurran, 1990) and coordinated hunting allows clans of spotted hyenas to more effectively hunt and defend large prey (Holekamp et al., 1997). Many bird species form remarkable flocks, often outside the context of breeding, that offer protection from predators and increase foraging efficiency (Powell, 1974; Sullivan, 1984). It has been proposed that such large social groups may be maintained by rewarding positive interactions with conspecifics and by reducing negative states caused by social separation (Emlen, 1952; Riters et al., 2019a). Moreover, within a flock optimal social spacing between conspecifics is important, indicating that individuals may optimize spacing to be close but not too close to conspecifics. Neuropeptides in the oxytocin and vasopressin family have been a major focus of research on nonsexual social bonding and flocking in birds (Goodson et al., 2009). However, opioids also underlie social behavior and are well known for both inducing reward and reducing negative affective states (Panksepp et al., 1980; Olds, 1982; Goeders et al., 1984). This suggests that opioids may play a central role in the maintenance of social groups.

Across species, μ-opioid receptor (MOR) stimulation facilitates nonsexual prosocial interactions. MOR activation is proposed to alleviate distress from maternal separation (Herman and Panksepp, 1978) and to reward social play (Normansell and Panksepp, 1990; Vanderschuren et al., 2016). Opioids are also strongly implicated in social interactions that promote cohesion in large groups, such as schools of fish (Kavaliers, 1981) and songbird flocks. In zebra finches, *Taeniopygia guttata*, administration of the nonselective opioid receptor antagonist naloxone suppresses undirected song—a type of song proposed to play a role in flock cohesion (Khurshid et al., 2010). In European starlings, *Sturnus vulgaris*, MOR stimulation facilitates singing behavior in flocks, referred to here as gregarious song, as well as the positive affective state associated with gregarious song (Stevenson et al., 2020). Additionally, gregarious singing behavior is associated with opioid-mediated analgesia (Kelm-Nelson et al., 2012). These findings together suggest that opioids released in association with social interactions may act at MORs to promote social cohesion by inducing reward and reducing the pain of being alone (Kelm-Nelson et al., 2012; Riters and Stevenson, 2012; Riters et al., 2014; Hahn et al., 2017; Stevenson et al., 2020).

Opioids act at MORs in numerous brain regions to induce reward and to alleviate negative affect. In songbirds, several studies demonstrate that the medial preoptic area [mPOA (commonly abbreviated as POM in birds)] is a crucial site in which MOR activation stimulates song in flocks and underlies song-associated reward (Riters et al., 2005, 2014; Stevenson et al., 2020). In mammals, the majority of studies on the role of MOR in reward, including nonsexual social reward (e.g., social play; Vanderschuren et al., 1997; Trezza et al., 2011; Manduca et al., 2016), focus on the nucleus accumbens (NAc), an integral part of the mesolimbic pathway that underlies motivation and reward (Salgado and Kaplitt, 2015). A few correlational studies have examined a potential role for the avian NAc in social behaviors such as pair bonding and female responses to male courtship (Svec et al., 2009; Earp and Maney, 2012; Riters et al., 2013), but none have tested the effects of experimental manipulations of MOR in the NAc on avian social behavior.

It could be that MORs in the avian mPOA are important for facilitating nonsexual social interactions in flocks, and a separate system involving MORs in the NAc underlies similar behaviors in mammals. However, the mPOA is part of a circuit that accesses the NAc through projections to the ventral tegmental area (Balthazart et al., 1994; Riters and Alger, 2004), suggesting that studies of social interactions in birds and mammals may be focusing on separate parts of an evolutionarily conserved circuity. Recent evidence in support of this idea comes from a study in juvenile rats that demonstrates a role for MORs in the mPOA in social play (Zhao et al., 2020). If MORs in the NAc are part of a conserved circuitry that stimulates social connections outside of a breeding context across vertebrates, then we predict that MOR stimulation in the NAc will facilitate prosocial behaviors in songbirds. To test this, we administered the selective MOR agonist d-Ala^2^-*N*-Me-Phe^4^-Glycol^5^-enkephalin (DAMGO) into the NAc of male and female European starlings (*S. vulgaris*) and measured vocal–social behaviors in nonbreeding flocks.

## Materials and Methods

### Animals and housing

All animal procedures were performed in accordance with the University of Wisconsin-Madison animal care committee regulations and adhere to National Institutes of Health guidelines.

Fourteen European starlings (*S. vulgaris*), eight males and six females, were included as experimental birds in this study. Additional birds were also included as unmanipulated flock mates to bring each flock to a total of either six or eight birds. The starlings were trapped from a local farm in Madison, Wisconsin and housed in same-sex cages with an 18 h/6 h light/dark cycle (lights on at 6:00 A.M.) until molting was complete. This photoperiod induces a condition referred to as “photo-refractoriness” that is characteristic of early fall when birds begin to sing in large mixed-sex flocks (Dawson et al., 2001). Birds were then housed with flockmates for the duration of the study in indoor aviaries (size: 2.13 × 2.4 × 1.98 m) that were decorated with tree branches and supplied with food, drinking water, and bathing water *ad libitum*. Talk radio was playing outside the aviaries during daylight hours to habituate birds to sounds outside the room.

This study was conducted from September 2019 through March 2020. After birds were tested and removed from the aviaries, new birds were added to each aviary. A single observer watched the flock daily to identify singing birds for inclusion in the study. During observation periods, audio recordings of starling song were played to facilitate singing behavior (Marius Travell, YouTube). Once singing birds were identified and selected, they underwent surgery to implant a cannula guide targeting the NAc (detailed below). A maximum of two birds from each aviary were selected to be tested on alternate days, and there was always at least one unmanipulated singing bird present in the aviary to facilitate flock song.

### NAc cannula surgery

**Figure 1.**
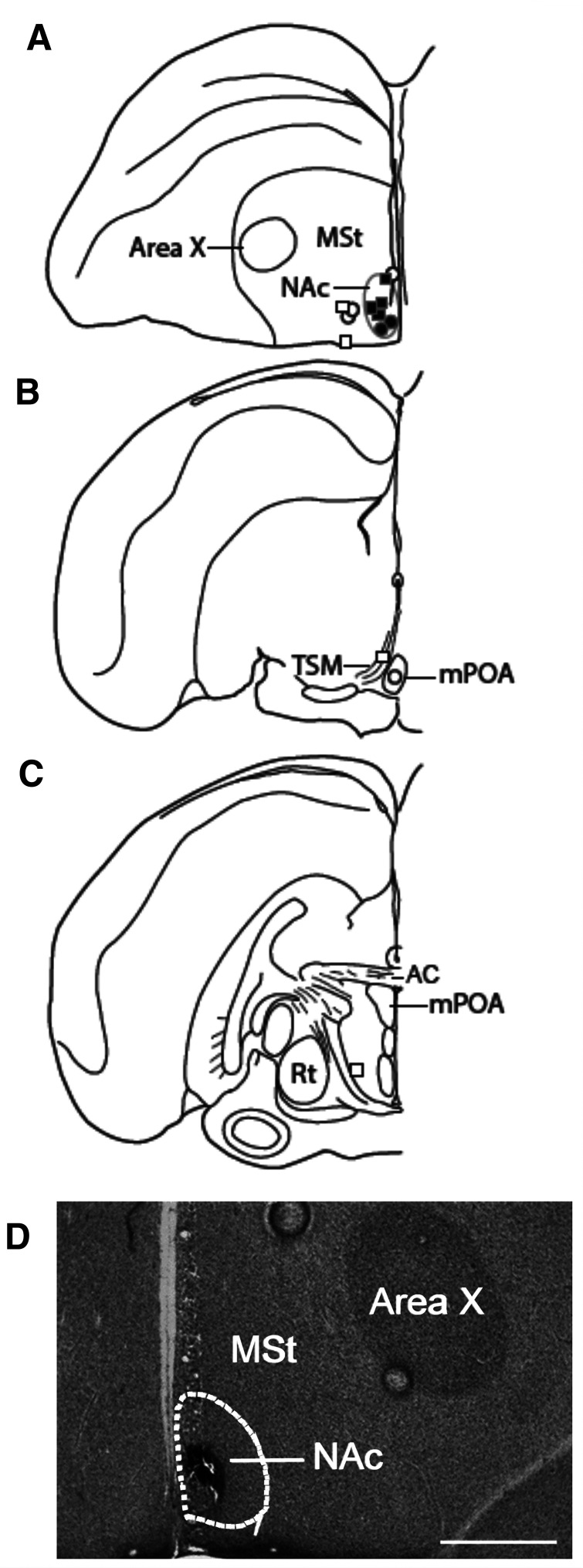
***A*–*C***, Location of DAMGO infusion sites. Illustration of one hemisphere of starling brain, with hits represented by filled-in shapes and misses represented by open shapes. Males are represented by squares, and females are represented by circles. ***D***, Photomicrograph of 2× magnification of a Nissl-stained brain section that demonstrates a hit with the cannula tip located in the NAc. Dotted line indicates boundaries of NAc. Scale bar, 500 μm (or 0.5 mm). AC, Anterior commissure; MSt, medial striatum; Rt, nucleus rotundus.

The cannula targeted the location of the songbird NAc proposed by Reiner et al. (2004). This region is in the rostral striatum, located medially surrounding the ventral tips of the lateral ventricles, which in our sections appears in coronal sections in which Area X is relatively small and round (Fig. 1). An 8 mm, 26 gauge cannula guide (C315G/SPC, Plastics One) was placed unilaterally into either the left or right NAc following procedures similar to those cited in the study by Kelm-Nelson et al. (2013). Birds were given an intramuscular injection of 0.10 ml of ketofen (Zoetis), anesthetized with isoflurane/oxygen gas (isoflurane: Patterson Veterinary; oxygen: Airgas), and secured in a stereotaxic apparatus (model 995, Kopf) using ear bars in the most rostral position of the ear, with the beak ∼45° below the plane of the ear bars. The dorsal head feathers were trimmed, and a small incision was made in the skin to visualize the skull. The cannula guide tip was laterally zeroed on the midvein, in a position 1.2 mm rostral to the ear bars. Then the guide was laterally rotated 4.5° and adjusted 1.0 mm to the left or the right, and vertical zero was again taken from the midvein. Three holes were drilled in the skull, one for the guide and two superficial holes for the screws. The guide tip was lowered ventrally 6.1 mm from the skull zero (the coordinate for dorsal NAc) and was secured using screws and instant dental cement (Ortho-Jet dental cement powder and acrylic liquid, Lang Dental Manufacturing). Once the dental cement was solidified, a dummy cannula (Plastics One) was fitted into the guide. The animal was then allowed to recover under monitoring until alert, then was transported and released into its home aviary. The birds were monitored that evening and again the following morning. Experimental treatments and observations began after the bird resumed singing in its flock, approximately 7 d after surgery.

### Pharmacological manipulations

The experimental birds were behaviorally tested on 4 d separated by at least 1 d. If two birds from an aviary were tested, they were tested on alternate days, such that only one bird per aviary was tested on a single day. A habituation treatment of vehicle (saline) was injected at least 2 d before the commencement of the treatment sequence to habituate birds to the procedure (details for the injection procedure are provided below). After this habituation injection, each treatment sequence lasted ∼3 weeks, consisting of the following four treatments: vehicle (sterile saline, 0.85%; 0.50 μl) and three doses of DAMGO (Sigma-Aldrich) [low dose, 0.025 μg; intermediate dose, 0.25 μg; or high dose, 2.5 μg (all dissolved in 0.50 μl sterile saline); catalog #100929–53-1]. Each bird received each treatment once in a counterbalanced order, and a minimum of 48 h separated treatments. The treatments were color coded by another researcher so that the observer would be blind to treatment conditions during all observations.

On each test day, injections began between 9:30 A.M. and 3:30 P.M. Experimental birds were swiftly caught in a net and anesthetized with isoflurane/oxygen using a nose cone. The cannula dummy was removed and replaced with a 33 gauge cannula connected to PE50 tubing (catalog #C232CT, Plastics One) containing the color-coded treatment solution. The cannula extended 2 mm beyond the tip of the cannula guide. A vacuum syringe (Hamilton) connected to a Nanomite Syringe Pump (Harvard Apparatus) injected 0.50 μl of the treatment solution over a 120 s period. The cannula was left in place for 180 s to allow for the equalization of pressure and diffusion from the cannula tip. Infusion volume was verified by following the movement of an air bubble in the tubing. The cannula was removed, the cannula dummy was replaced, and the bird was placed into a draped recovery cage for 15 min before being released into its home aviary.

### Behavioral observations

A single researcher observed each focal animal using a continuous sampling technique. The following behaviors were recorded for 40 min: social approaches (the focal individual approaches to within 10 cm of another bird and remains near that bird); displacements (the focal individual approaches to within 10 cm of another bird followed by the receiving bird leaving proximity within 1 s); gregarious singing (sum of introductory whistles and song bouts separated by at least 1 s); calls (sum of calls; however, we did not identify specific call types); perch changes (sum of movement along or between branches separated by at least 1 s); beak wipes (beak wipes in starlings are not used as part of courtship and are considered to be a sign of stress; Bauer et al., 2011); and feeding (sum of bouts of feeding separated by at least 1 s or a complete head lift). After the first 20 min observation period, a high-reward food (i.e., horse feed, which attracts large flocks in local barns) was added to examine the possibility that MOR stimulation in NAc would stimulate hedonic feeding, as observed in rats (Bakshi and Kelley, 1993; Zhang et al., 1998); however, this food was ignored by the birds and will not be discussed further.

### Cannula tip verification

Following the final treatments and observations, the birds were injected with 1.0 μl of Chicago Blue 6B dye (Thermo Fisher Scientific) or with a tract tracer using the same injection procedure as above, to identify the location of the tip of the cannulae. For the birds infused with blue dye, after infusion the birds were rapidly decapitated, and their brains were removed and frozen on crushed dry ice. The brains were stored at −80°C and then sectioned at 50 μm using a cryostat (catalog #CM1850, Leica Biosystems). Sections were mounted on slides, dehydrated, Nissl stained, and coverslipped. The slides were analyzed under a microscope to determine whether the blue dye was contained within the NAc (“hit”) or elsewhere (“miss”). For three birds, 0.20 μl of the neuronal tracer biotinylated dextran amine (molecular weight, 3000; lot #2089930, NeuroTrace) at 10% concentration was infused to confirm the site of injection (these infusions were part of a study not reported here). Ten days after infusion of the tracer, birds were perfused and the tracer was visualized as described in the study by Riters and Alger (2004). Sex and nonbreeding condition were verified immediately following death via the presence and size of testes.

### Statistical analysis

Statistical analysis was conducted using GraphPad Prism for Windows (version 8.0.0; GraphPad Software; www.graphpad.com). The proportion of each behavior produced on each day was used for analysis to control for high levels of individual variation in behavior across birds (Table 1, untransformed descriptive statistics). Specifically, for each bird and each behavioral measure, we took the sum of that behavior for each test day and divided it by the total sum of that behavior for all days. Difference scores between behavior frequency after treatments and behavior frequency under saline were also examined, and results were consistent with proportions of behavior; here we report the proportions. A general linear model (GLM) was run for each behavior, with treatment entered as repeated measures, cannula location entered as a factor, and proportion of behavior entered as response variables, with separate analyses run for each behavior. Tukey’s HSD *post hoc* tests were run following significant (*p* < 0.05) GLM results. Some of the misses targeted areas in which we expected MORs to regulate social behavior (see Discussion), which reduced the likelihood of seeing a significant interaction, potentially precluding *post hoc* comparisons of doses within treatment groups. However, the goal of this study is to characterize the role of MOR specifically in the NAc in social behaviors. Thus, to provide additional insight, in cases where there was a dose effect but no dose × cannula interaction, we ran exploratory Dunnett’s *post hoc* analyses on hits only to compare the effects of the most effective dose of agonist (i.e., the highest dose) against the other treatments. A Levene’s test was run to test the assumption of homogeneity of variance, and a Lilliefors test was run to test the assumption of normality. When assumptions were violated, analyses were run on log-transformed data. For instances where there were zero occurrences of a behavior, a formula of log(*x *+* *0.05) was used. When transformation did not correct the violation of assumptions, nonparametric Friedman’s ANOVAs were used to examine treatment differences for hits and for misses. Effect sizes were calculated using η^2^. Confidence intervals at 95% were determined using the untransformed proportions of each behavior. Pearson correlations for all behaviors at baseline (saline) were run to examine potential relationships between behaviors.

**Table 1:** Untransformed behavior measurements for each treatment and condition

Behavior	Cannula	Dose			
		Saline	0.025 μg DAMGO	0.25 μg DAMGO	2.5 μg DAMGO
Singing	Hit	0 ± 0	0 ± 0	0 ± 0	26.17 ± 15.86
	Miss	0 ± 0	1.25 ± 1.25	0.13 ± 0.13	0.25 ± 0.25
Calls	Hit	42.17 ± 26.5	10.67 ± 5.47	32 ± 23.2	134.67 ± 65.2
	Miss	21.75 ± 12.23	17.5 ± 9.31	21.38 ± 10.91	28.63 ± 8.55
Approaches	Hit	1.83 ± 1.05	2.33 ± 1.17	3.33 ± 0.84	5.50 ± 1.34
	Miss	4.88 ± 2.96	3.88 ± 1.23	2.63 ± 0.96	4.63 ± 1.41
Displacements	Hit	9.33 ± 2.26	8.83 ± 2.27	10.67 ± 4.17	28.5 ± 6.53
	Miss	6.25 ± 1.46	7 ± 2.17	5.25 ± 1.71	9.63 ± 2.68
Perch changes	Hit	87.17 ± 21.89	105.67 ± 17.86	158.67 ± 33.05	359.67 ± 74.1
	Miss	197 ± 65.05	132.25 ± 15.01	124.25 ± 22	245.25 ± 52.64
Feeding	Hit	6.67 ± 3.3	9.67 ± 1.8	12.17 ± 5.99	16 ± 5.94
	Miss	6.5 ± 1.87	5.63 ± 1.15	3.5 ± 1.13	10.38 ± 1.81
Beak wipes	Hit	28.5 ± 8.25	34.83 ± 7.32	33 ± 3.51	34.33 ± 8.28
	Miss	26 ± 6.63	40.63 ± 16.78	30.38 ± 7.76	38.88 ± 5.99

Values are the mean ± SEM.

## Results

The cannula tip was contained within the bounds of the NAc in six birds (hits; four males, two females) and outside the NAc in eight birds (misses; four males, four females; Fig. 1). For the misses, the cannula tips were contained within the striatum lateral to NAc (*n* = 4), the lateral hypothalamus (LH; *n* = 1), the mPOA (*n* = 1), and the tractus septopallio-mesencephalicus (TSM; *n* = 1), the ventricle (*n* = 1). There was not adequate statistical power to analyze sex, but results indicate that the same trends were observed in males and females, so males and females were combined, but figures indicate males and females (Stevenson et al., 2020).

### Effects of MOR stimulation on social behaviors

#### Singing behavior

**Figure 2.**
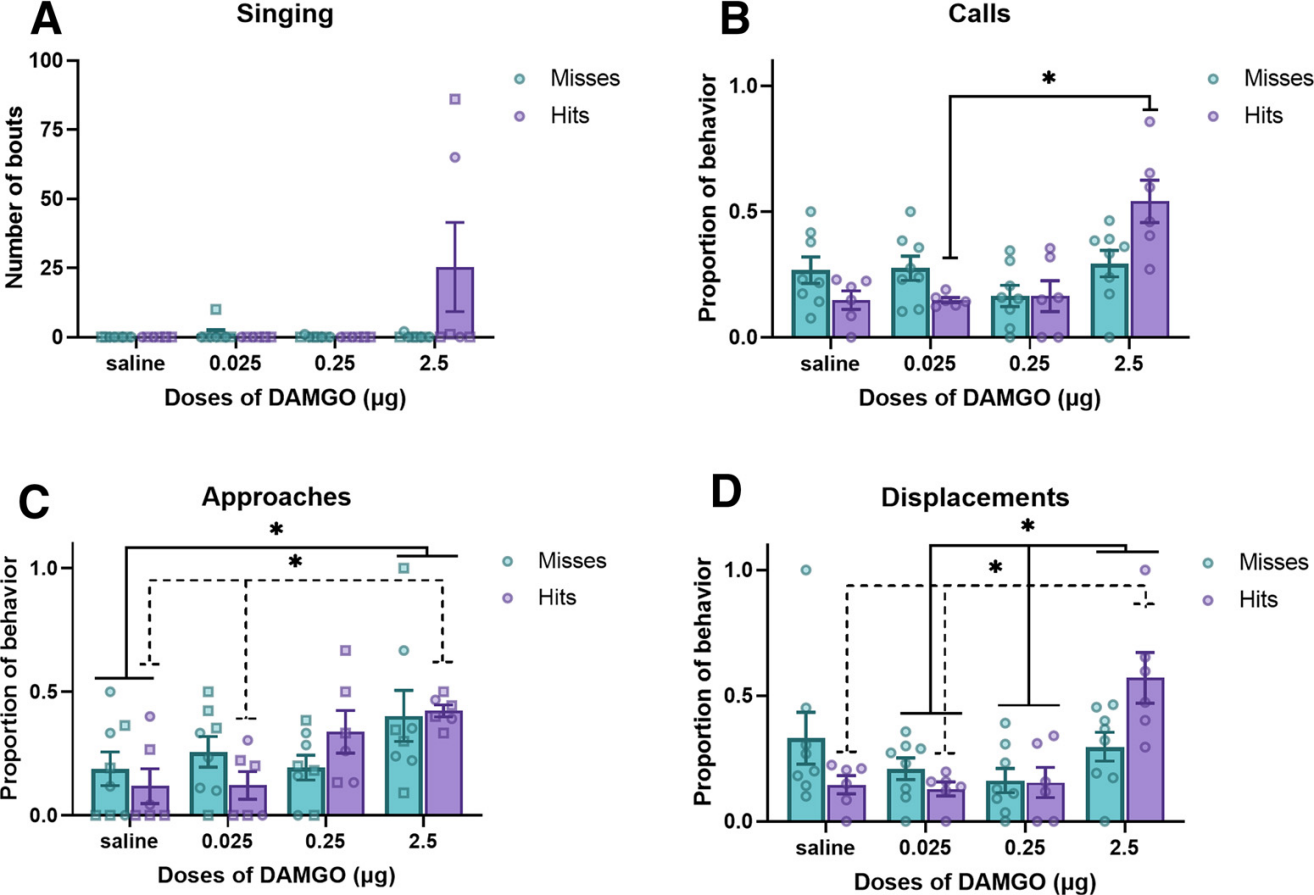
Effects of MOR stimulation in NAc on song, calls, social approaches, and displacements (mean ± SEM). ***A–D***, The number of song bouts (***A***), number of calls (***B***), proportion of approaches (***C***), and proportion of displacements (***D***) in male (squares) and female (circles) starlings in which the cannula tip missed the NAc (*n* = 8) or hit the NAc (*n* = 6). Analyses were run on log-transformed data for both ***C*** and ***D*** to correct for assumptions, but here we show untransformed proportions. Dotted lines represent pairwise significance for intra-NAc dose effect. *p < 0.05, **p < 0.01.

Statistics were not conducted on singing behavior because most birds did not sing, driving variance down to zero for most doses (Fig. 2*A*, Table 1, untransformed descriptive statistics). Of six hits, three birds (50%; two males, one female) sang when treated with the highest dose of MOR agonist. Of eight misses, only two birds sang at any dose (one male, one female). The male sang at the lowest dose of the MOR agonist (0.025 μg DAMGO), and the cannula tip was located in the striatum lateral to NAc. The female sang at the highest dose (2.5 μg DAMGO), and the cannula tip was located in the mPOA.

#### Calling behavior

For calls, a GLM revealed a significant main effect for dose (*F*_(3,36)_ = 6.95, *p* = 0.013, η^2^ = 0.317). No significant main effect was found for cannula location (*F*_(1,12)_ = 1.37, *p* = 1.0, η^2^ = 0.0), but there was a significant dose × cannula location interaction (*F*_(3,36)_ = 3.32, *p* = 0.003, η^2^ = 0.188). *Post hoc* analysis revealed that birds with a cannula tip in the NAc treated with the highest dose of MOR agonist called significantly more than when treated with the lowest dose of MOR agonist (*p* = 0.023), but not with saline (*p* = 0.064) or the intermediate dose of MOR agonist (*p* = 0.15). There were no significant differences found between any dose for misses [*p* > 0.05 for all comparisons; Fig. 2*B* (also see Fig. 4*A*), Tables 1, 2, untransformed descriptive statistics and confidence intervals].

**Table 2:** 95% Confidence intervals based on the proportions of the occurrences for each behavior and condition

Behavior	Cannula	Dose			
		Saline	0.025 μg DAMGO	0.25 μg DAMGO	2.5 μg DAMGO
Approaches	Hit	[−0.02, 0.256]	[0.011, 0.231]	[0.169, 0.507]	[0.375, 0.47]
	Miss	[0.055, 0.322]	[0.137, 0.38]	[0.062, 0.24]	[0.2, 0.605]
Calls	Hit	[0.038, 0.269]	[−0.004, 0.185]	[−0.012, 0.371]	[0.264, 0.889]
	Miss	[0.053, 0.348]	[0.006, 0.345]	[0.058, 0.293]	[0.242, 0.656]
Displacements	Hit	[0.053, 0.238]	[0.058, 0.201]	[0, 0.309]	[0.311, 0.83]
	Miss	[0.088, 0.575]	[0.108, 0.311]	[0.049, 0.276]	[0.162, 0.432]
Perch changes	Hit	[0.06, 0.194]	[0.12, 0.18]	[0.116, 0.341]	[0.371, 0.616]
	Miss	[0.279, 0.324]	[0.168, 0.253]	[0.12, 0.246]	[0.258, 0.452]
Feeding	Hit	[0.097, 0.156]	[0.14, 0.421]	[0.132, 0.347]	[0.238, 0.471]
	Miss	[0.118, 0.383]	[0.137, 0.307]	[0.041, 0.222]	[0.269, 0.523]
Beak wipes	Hit	[0.139, 0.226]	[0.159, 0.369]	[0.132, 0.361]	[0.242, 0.372]
	Miss	[0.097, 0.313]	[0.112, 0.445]	[0.162, 0.368]	[0.14, 0.363]

#### Social approaches

A GLM run on the log-transformed approach data revealed a significant main effect for dose (*F*_(3,36)_ = 4.84, *p* = 0.007, η^2^ = 0.233). No significant main effect was found for cannula location (*F*_(1,12)_ = 0.17, *p* = 0.689, η^2^ = 0.001), and there was no dose × cannula location interaction (*F*_(3,36)_ = 1.84, *p* = 0.157, η^2^ = 0.089). *Post hoc* analysis of the dose effect revealed that birds treated with the highest dose of MOR agonist approached significantly more often than birds treated with saline (*p* = 0.019). *Post hoc* analysis run on the hits demonstrates that the highest dose of intra-NAc MOR agonist was significantly higher than that of saline (*p* = 0.032) and the lowest dose (*p* = 0.034), but not the intermediate dose, of MOR agonist [*p* = 0.523; Fig. 2*C* (also see Fig. 4*B*), Tables 1, 2].

#### Displacements

A GLM run on the log-transformed sum of displacements revealed a significant main effect for dose (*F*_(3,36)_ = 5.33, *p* = 0.009, η^2^ = 0.248). No significant main effect was found for cannula location (*F*_(1,12)_ = 0.14, *p* = 0.712, η^2^ = 0.001) or the dose × cannula location interaction (*F*_(3,36)_ = 2.27, *p* = 0.097, η^2^ = 0.106). *Post hoc* analysis of the dose effect revealed that birds displayed a significantly higher proportion of displacements when treated with the highest dose of MOR agonist than when treated with the low dose (*p* = 0.015) or intermediate dose (*p* = 0.034) of MOR agonist. *Post hoc* analysis run on the hits demonstrates that the highest dose of intra-NAc MOR agonist yielded a significantly higher proportion of displacements than saline (*p* = 0.040) and the lowest dose (*p* = 0.022) but not the intermediate dose (*p* = 0.088) of MOR agonist [Fig. 2*D* and (also see Fig. 4*C*), Tables 1, 2].

### Effects of MOR stimulation on nonsocial behaviors

#### Perch changes

**Figure 3.**
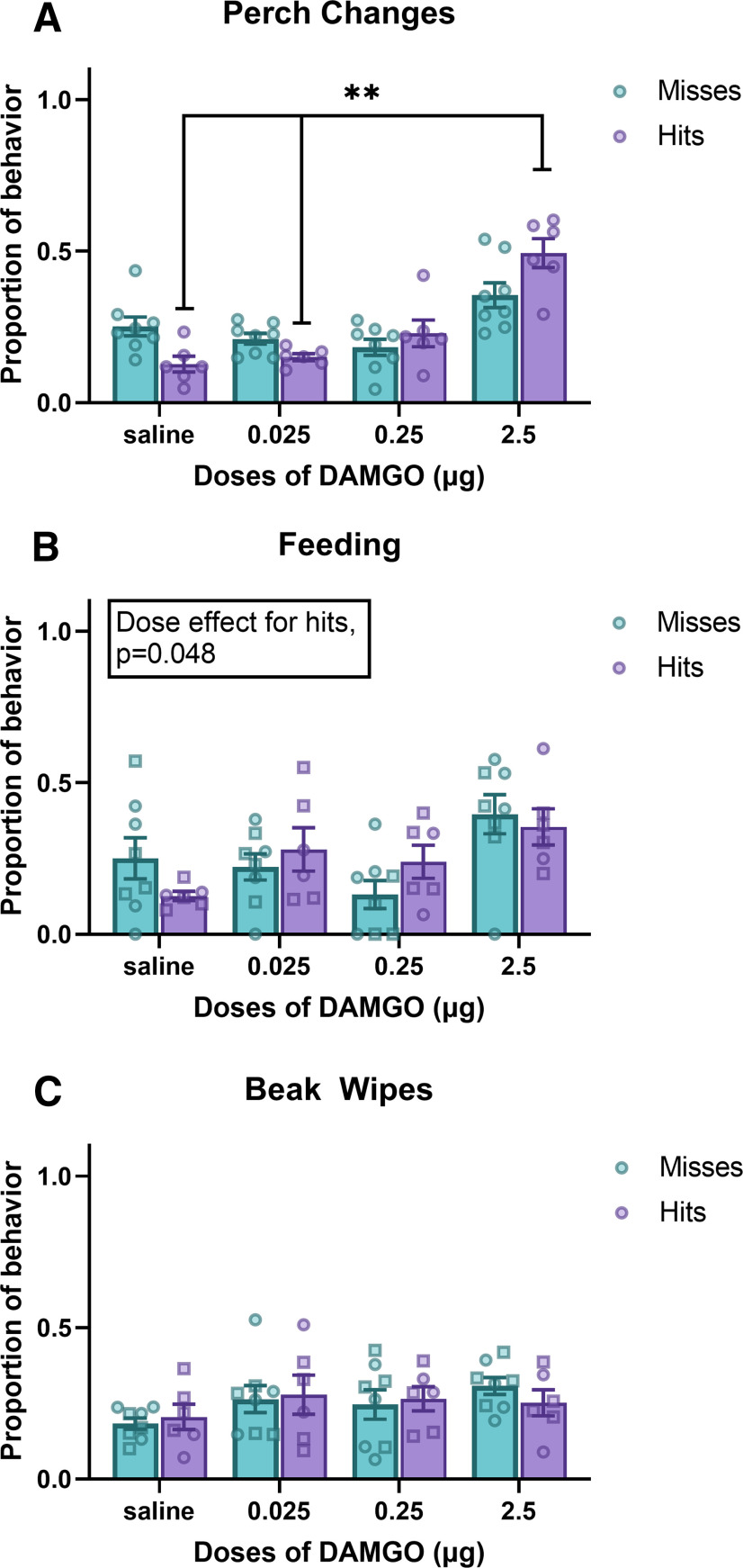
Effects of MOR stimulation in NAc on nonsocial behaviors (mean ± SEM). ***A–C***, Proportion of perch changes (***A***), feeding (***B***), and beak wipes (***C***), in male (squares) and female (circles) starlings in which the cannula tip missed the NAc (*n* = 8) or hit the NAc (*n* = 6). *p < 0.05, **p < 0.01.

**Figure 4.**
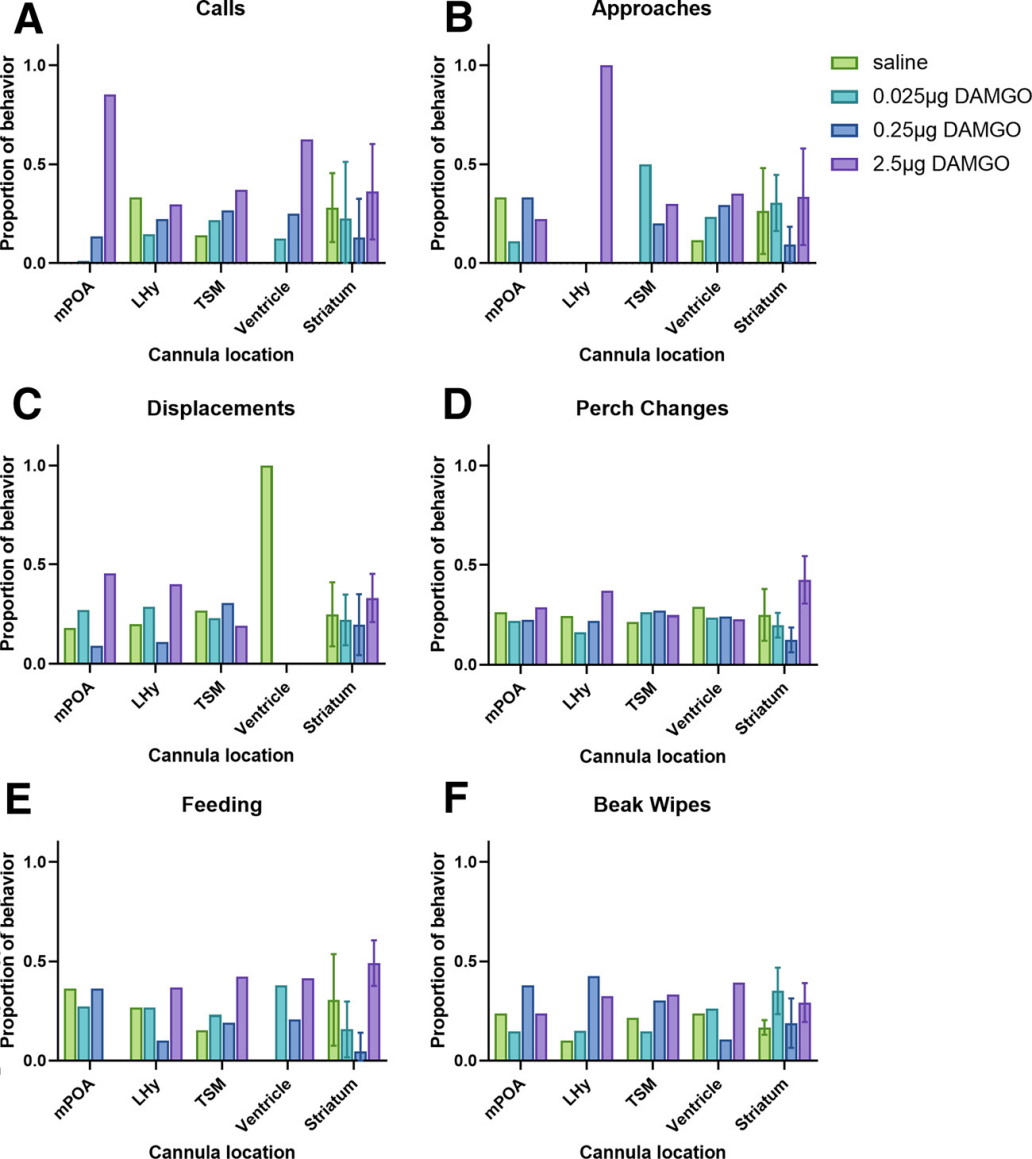
Effects of MOR stimulation in locations in which the cannula tip was located outside NAc (mean ± SEM). ***A–F***, Proportion of calls (***A***), social approaches (***B***), displacements (***C***), perch changes (***D***), feeding (***E***), and beak wipes (***F***) in birds with cannula located in the mPOA, TSM, lateral ventricle, and striatum lateral to NAc (Fig. 1). *n* = 1 for each location except for the striatum (*n* = 4) where the mean ± SEM is given.

For perch changes, a GLM revealed a significant main effect for dose (*F*_(3,36)_ = 18.94, *p* < 0.001, η^2^ = 0.560). No significant main effect was found for cannula location (*F*_(1,12)_ = 2.56, *p* = 0.135, η^2^ = 0.0), but there was a significant dose × cannula location interaction (*F*_(3,36)_ = 4.70, *p* = 0.007, η^2^ = 0.139). *Post hoc* analysis revealed that birds with a cannula tip in the NAc treated with the highest dose of MOR agonist had a significantly higher proportion of perch changes than when treated with saline (*p* = 0.008) or the lowest dose of MOR agonist (*p* = 0.003), but not the intermediate dose of MOR agonist (*p* = 0.103). There were no significant differences found between any dose for misses (*p* > 0.05 for all comparisons; Figs. 3*A*, 4*D*, Tables 1, 2).

#### Feeding

A transformation of feeding data did not correct assumptions needed to run parametric statistics. Nonparametric Friedman’s ANOVA tests yielded a significant effect for dose for hits (ANOVA, χ^2^ test = 7.48 (*n* = 6, df = 3, *p* = 0.048), but not misses [ANOVA, χ^2^ test = 7.78 (*n* = 8, df = 3, *p* = 0.051); Figs. 3*B*, 4*E*, Tables 1, 2]. *Post hoc* analysis yielded no significant differences between doses for either hits or misses (*p* > 0.05 for all comparisons), although for hits the highest dose compared with saline was *p* = 0.06.

#### Beak wipes

For beak wipes, a GLM revealed a significant main effect for cannula location (*F*_(1,12)_ = 7.71, *p* = 0.017, η^2^ = 0.0). No significant main effect was found for dose (*F*_(3,36)_ = 1.26, *p* = 0.304, η^2^ = 0.092) or the dose × cannula location interaction (*F*_(3,36)_ = 0.29, *p* = 0.831, η^2^ = 0.020; Figs. 3*C*, 4*F*, Tables 1, 2). However, *post hoc* analysis yielded no significant difference between hits and misses at any dose (*p* > 0.05 for all comparisons).

### Correlation of behaviors

In addition, correlations were run for all behaviors listed above to determine whether the base levels of any behavior correlated with another behavior, possibly indicating that an increase in one would drive an increase in another. However, we found no evidence of any correlations between these behaviors (*p* > 0.05 for all comparisons; Table 3).

**Table 3:** Correlation matrix for behavioral proportions at baseline (saline) in combined hits and misses

	Approaches	Displacements	Perch changes	Feeding	Calls	Beak wipes
Approaches	1.0					
Displacements	0.36	1.0				
Perch changes	0.08	0.41	1.0			
Feeding	−0.06	0.24	0.17	1.0		
Calls	0.05	0.35	0.52	0.18	1.0	
Beak wipes	−0.09	0.28	−0.24	−0.05	0.26	1.0

*r* Values are shown. All *p* > 0.05.

## Discussion

This study is the first to demonstrate a causal role for the songbird NAc in the facilitation of social interactions in nonsexual gregarious contexts. Results demonstrate a role for MOR in NAc in behaviors considered important for group cohesion, including gregarious song, social approach, and displacements, with similar patterns observed in males and females. More broadly, these data provide evidence that the NAc may be part of a conserved circuitry that promotes social cohesion in nonsexual contexts across vertebrates.

### MOR stimulation in the NAc increased social-spacing behaviors

The highest intra-NAc dose of the MOR agonist DAMGO facilitated social approach behaviors, offering support for a role for MOR in the NAc in prosocial behavior. Results of past studies in rodents also demonstrate a role for MORs in social proximity; however, in these studies peripherally administered MOR agonists decreased the amount of time rodents spent near conspecifics (Herman and Panksepp, 1978; Panksepp et al., 1979). Although the findings were in the opposite direction from our study, these past results were interpreted to suggest that the agonist replaced the need for reward that would normally be induced by opioids released by social contact (Herman and Panksepp, 1978). Indeed, many rewarding behaviors [e.g., social play (Trezza et al., 2010; Achterberg et al., 2019) and hedonic feeding (Berridge, 2009; Gosnell and Levine, 2009)] are facilitated by low doses and inhibited by higher doses of MOR agonists. This suggests that the dose of agonist and site-specificity in our study may have been sufficient to facilitate social behavior, but not sufficient to fully replace social reward. Future studies are needed to test this hypothesis.

The highest intra-NAc dose of the MOR agonist DAMGO also stimulated displacements, a mildly agonistic behavior. This is similar to past findings that show that peripheral administration of a MOR agonist facilitates agonistic behaviors in response to an intruder in mice (Campbell Teskey and Kavaliers, 1988); however, relative to territorial defense, the agonistic interactions in the present study were relatively nonthreatening. Starlings do not maintain strong, linear dominance hierarchies when in large flocks and are often observed sharing food sources. Therefore the function of displacements does not appear to relate to dominance, but may play a role in the maintenance of adequate social spacing (King, 1973). Starlings may space themselves to maintain optimal levels of natural MOR stimulation, and infusion of a MOR agonist may slightly reduce the need for close social contact, as previously stated, perhaps triggering mildly agonistic interactions to maintain social spacing. The finding that the activation of MORs in the NAc stimulates approach while at the same time increasing behaviors used to maintain social spacing suggests that MOR in the NAc may play a role in optimizing the pull of joining the flock with the push of potential agonistic encounters.

### MOR stimulation may stimulate vocal behaviors

Although few birds sang, the administration of the highest intra-NAc dose of the MOR agonist DAMGO initiated gregarious singing behavior in 50% of birds tested. Song in this context is highly sensitive to stressors, and when a bird is caught and injected, it stops singing (Stevenson et al., 2020); therefore, restoring singing behavior with intra-NAc MOR stimulation is noteworthy. This type of gregarious singing behavior in a nonbreeding context is associated with an intrinsic reward state (Kelm-Nelson et al., 2012; Riters and Stevenson, 2012; Riters et al., 2014; Hahn et al., 2017; Stevenson et al., 2020) and is proposed to be a form of play behavior that allows birds to develop important social skills for use in more serious reproductive contexts (Riters et al., 2019b). MOR stimulation in the NAc also dramatically stimulates and rewards social play in rodents (Vanderschuren et al., 1997; Trezza et al., 2011; Manduca et al., 2016). Thus, the present study is the first to implicate MOR in the NAc in gregarious song, and it provides neuropharmacological support for the hypothesis that playful behaviors involve neural systems that are conserved across vertebrates.

In addition, the highest intra-NAc dose of the MOR agonist DAMGO stimulated calls. Functionally distinct calls are used by starlings in flocks (Feare, 1984); however, we did not distinguish between different call types. Therefore, although we can conclude that MOR stimulation increased calls in this study, future studies are needed to explore the functional role of opioids in calling behavior.

### MOR stimulation of nonsocial behaviors

This is the first study to examine the effects of MOR agonist administration in the NAc of songbirds. The present study focused on the location of the songbird NAc proposed by Reiner et al. (2004). The functional homology of this site to the mammalian NAc has not been well studied. To both determine the degree to which our focal area is functionally similar to the mammalian NAc and to explore the specificity of effects on social behavior, we measured motor activity, feeding, and stress-related behaviors—all behaviors that are influenced by MOR agonist infusion into the NAc in rodents.

The highest intra-NAc dose of the MOR agonist DAMGO increased motor activity, as reflected in perch changes. Perch changes did not correlate with either approaches or social-spacing behaviors, therefore we do not consider motor activity to be driving the increase seen in either type of behavior. This increase in motor activity is consistent with studies that show similar effects in rodents (Vezina et al., 1987; Cunningham and Kelley, 1992; Bakshi and Kelley, 1993). Intra-NAc MOR stimulation also increased feeding, with the high dose, when compared with saline, being just shy of significance (*p* = 0.06). Several studies in rats demonstrate that intra-NAc DAMGO administration most powerfully increases hedonic feeding, including the consumption of highly palatable, high-fat food (Bakshi and Kelley, 1993; Zhang et al., 1998; Will et al., 2003). Therefore, it is possible that if the present study examined effects on a highly palatable food option, there may have been a more substantial increase in feeding behavior. Although peripheral MOR administration alters stress responses, the present data found that administration of MOR agonist in the avian NAc had no impact on beak wipes, which are considered an indication of stress in starlings (Bauer et al., 2011). These data suggest that opioids may act on MOR in other brain regions outside the NAc in flocking songbirds to reduce stress.

### Effects of MOR stimulation outside NAc

Although birds with the cannula located outside the NAc were considered controls, for some of these birds the cannula tips were located in brain regions known to influence social and other behaviors. Sample sizes in most cases consist of a single bird, so individual cases are not analyzed; rather, a few cases are highlighted here that are consistent with past research. Specifically, for one of the birds the cannula tip was located in the mPOA. For this bird, the highest dose of the MOR agonist DAMGO stimulated gregarious song, which is consistent the findings of a recent study that showed that downregulation of MOR in the starling mPOA reduces gregarious singing (Stevenson et al., 2020). The highest dose of the MOR agonist DAMGO also stimulated feeding in birds with the cannula tip located in the striatum with a similar result observed for a bird with the tip located in the lateral hypothalamus, results that are similar to findings in rodents (Levine and Billington, 1989; Zhang and Kelley, 2000; Castro et al., 2015; Ardianto et al., 2016). The finding that displacement behaviors were higher for birds receiving high doses of MOR agonist in the lateral hypothalamus and mPOA is consistent with studies that implicate these regions in agonistic behavior (Panksepp, 1971; Hammond and Rowe, 1976; Schlinger and Callard, 1990; Zhang and Kelley, 2000; Nieh et al., 2016). These results suggest sites in which MOR may act to influence these behaviors; thus, the misses support past research and suggest potential roles for MOR in areas outside the NAc in social and nonsocial behaviors that can be tested in future research.

### Conclusions

The results of this study suggest that a region identified as the NAc in birds (Reiner et al., 2004) is functionally homologous to the mammalian NAc, and that this region may be part of a core, conserved circuitry that underlies rewarding social behaviors across vertebrates. Future studies are now needed to examine proposed shell and core subdivisions of NAc in birds, which in mammals play distinct roles in reward (Kelley, 1999), as well as the extent to which the mesolimbic reward pathway is differentially involved in modulating social spacing behaviors seasonally in songbirds.

## Synthesis

Reviewing Editor: Karyn Frick, University of Wisconsin-Milwaukee Decisions are customarily a result of the Reviewing Editor and the peer reviewers coming together and discussing their recommendations until a consensus is reached. When revisions are invited, a fact-based synthesis statement explaining their decision and outlining what is needed to prepare a revision will be listed below. The following reviewer(s) agreed to reveal their identity: Scott MacDougall-Shackleton.

The manuscript by Maksimoski et al. demonstrates that mu opioid receptors (MORs) in the nucleus Accumbens (NAc) are important for affiliative behaviors and to maintain social spacing. The reviewers felt that the authors have performed a well-designed study to investigate whether MORs in the NAc are involved in maintaining social connections in non-breeding flocks of European starlings. Because MORs in NAc are considered to underlie reward and the reduction of negative affective states, they have tested whether stimulating these receptors by injecting the MOR agonist DAMGO in NAc in non-breeding starlings of both sexes lead to changes in their social interactions. Their results suggest a dose-dependent increase in singing, social approaches, as well as mild agonistic behaviors for social spacing following infusions of DAMGO in NAc of both male and female birds. Their findings suggest that MOR in NAc are important for promoting social interactions while simultaneously maintaining social spacing for maintaining the flock in a non-breeding context.

Because earlier studies have shown similar functions for MORs in the rodent NAc, the reviewers agreed that the data significantly advance the field by suggesting homologous circuits between birds and mammals that are conserved across evolution. Moreover, the reviewers felt the paper to be very clear, well written, and well conducted, with explicitly stated objectives. In the words of one reviewer, “It is a beautiful piece of work”.

However, the reviewers did have some concerns that they felt must be addressed as follows:

Major Comments:

1. The authors summed behaviors within categories. The statistics they ran are fine, but summing to provide the data is not. When you sum, let’s say, 3 different behaviors , that gives each behavior equal weight. So a bird with score 3 may have two flights and a hop, and another bird with a score 3 may have 3 hops, and they are inappropriately treated as equivalent. The ‘raw’ data are not actually included in Table 1 as claimed. It just has the means and SEM. The raw data should be included or deposited online. The correct analysis here would be to combine the raw measures with a factor analysis, like principal components, and then run the GLM on the scores that are output from the factor analysis. Simply summing the behaviors is not statistically valid.

2. The authors have mentioned that experimental birds were behaviorally tested on 4 days separated by at least one day but they need to clarify whether each experimental bird was only tested once with each dose of DAMGO. Were the doses of DAMGO randomized across trials? How soon is DAMGO metabolized in starlings? Is there any possibility of cumulative effects of DAMGO across trials?

3. Other than song, were there any differences in the kinds of calls that the birds produced?

4. The authors suggest that social approach and social spacing may be part of the same behavior. While they have provided a good description of both, they should provide video recordings of these behaviors for further clarity.

5. Beak-wipes are important for grooming and courtship in most birds - the authors should include references of their being a sign of stress in starlings.

6. The results demonstrate that the increase in motor activity following DAMGO injections in NAc was not correlated with social approach or social spacing. Since both involve movement, this is difficult to understand and needs further clarification.

Minor Points:

7. Statistical Analysis: The sentence ‘Thus to provide additional insight, in cases where there was a dose effect but no dose-by-cannula interaction, we ran exploratory Dunnett’s post hoc analyses ...... ‘ is incomplete - an ‘of’ is missing here.

8. Discussion: In the section on ‘MOR stimulation of non-social behaviors’ (second paragraph):

The sentence ‘Several studies in rats demonstrate that intra-NAc DAMGO administration most powerfully increases consumption of hedonic feeding including highly palatable, high fat food’ should be replaced by:

‘Several studies in rats demonstrate that intra-NAc DAMGO administration most powerfully increases hedonic feeding including the consumption of highly palatable, high fat food’

9. Conclusions: Typo in the first sentence - The results of ‘his’ study should be ‘this’ study..
